# Mating disruption of the tomato leafminer *Tuta absoluta* (Lepidoptera: Gelechiidae) on greenhouse tomatoes

**DOI:** 10.1007/s44297-024-00035-y

**Published:** 2024-09-27

**Authors:** Junxia Huang, En Wu, Chunxi Yang, Xiangyu Han, Jinghang Zhang, Mengyu Cao, Fengzhi Deng, Qianshuang Guo, Yongjun Du

**Affiliations:** 1 Inner Mongolia Autonomous Region Plant Protection and Quarantine Center, Hohhot, 010010 China; 2 Chifeng Agricultural and Animal Husbandry Technology Extension Center, Chifeng, 024000 China; 3 Chifeng Ningcheng County Plant Protection Station, Chifeng, 024200 China; 4 Institute of Pesticides and Environmental Toxicology, Zhejiang University, Hangzhou, 310058 China

**Keywords:** *Tuta absoluta*, Mating disruption, Sex pheromone dosage, Dispenser

## Abstract

Mating disruption is an important component of the integrated management system for the tomato leaf miner *Tuta absoluta* (Meyrick). This study showed that the dosage in the tubing dispensers is closely related to their attractiveness. *E*3*Z*8*Z*11-14:Ac and the binary mixture of *E*3*Z*8*Z*11-14:Ac and *E*3*Z*8-14:Ac at a dosage of 80 mg and a placement density of 900 polyethylene (PE) tube dispensers/ha significantly reduced the numbers of *T. absoluta* adults, larvae and damaged tomato leaves. When the adult density was low in the greenhouse, the relationship between the placement density of dispensers (*x*) and the number of moths caught (*y*) was y=310.6-1.06*x*+0.0008*x*^2^, which is a typical competitive mating disruption. However, when the adult density was high, the regression equation was* y*=-1.112*x*+959.4. Both passive and active dispensers significantly reduced the damage rate and larval population, but the 900 tube dispensers/ha and active aerosol dispensers had the best control results. The effects of the competitive passive dispensers and the placement density of dispensers were influenced by the adult density. At high density, 900 tube dispensers/ha were required.

## Introduction

The tomato leafminer *Tuta absoluta* (Meyrick) (Lepidoptera: Gelechiidae) is a worldwide pest of tomatoes. Due to global climate change, suitable temperatures are conducive to its development and survival [1–3]. It has spread from South America to Europe, South Asia, and Central Asia. In China, it was first detected in 2017 and has so far been found in 21 provinces [4–6]. *T. absoluta* adult has a long lifespan with high reproductive potential, while the larva tunnels tomato fruit, leaf and stem to cause damage [5, 6]. The management of this pest has been heavily dependent on the application of synthetic insecticides. Because of the larva protected inside the plant and the insecticide resistance, chemical control often fails but may cause environmental and agricultural product safety issues [7, 8]. Therefore, other components such as insect sex pheromone technology in the integrated pest management (IPM) should be considered [9–11]. The sex pheromone of *T. absoluta* has been identified as trans-3-cis-8-cis-11-tetradecatriene acetate (*E*3*Z*8*Z*11-14:Ac) [12, 13] with trans-3-cis-8-tetradecadiene acetate (*E*3*Z*8-14:Ac) as a trace component [13], and its activity has been demonstrated in field trials [13–15]. The specificity and trapping with *T. absoluta* sex pheromones are intuitive. However, the males can only be trapped by sticky or water basin trap, which requires frequent replacement of the sticky card or addition of water [11]. Obviously, both of them are not feasible for mass trapping in the field. *T. absoluta* adults can mate soon after their emergence. When the population density is high enough, the male and female adults can mate nearby without the sex pheromone attraction. In addition, the testicular volume of the males caught by the sex pheromone trapping was relatively smaller, equivalent to those in males aged 7 to 11 days [16]. Therefore, mass trapping is not feasible for the control of this pest [17].

Mating disruption (MD) involves releasing a high dosage of insect sex pheromone odor through a dispenser, causing the pheromone plume to spread everywhere in the field to interfere with the calling and mating of moths. It has been widely used in the IPM systems [18]. Currently, there are two main types of dispensers for MD: one is active and the other is passive. Active dispensers uses a mechanical spray system to control the release timing and the amount of sex pheromones based on the calling and mating rhythms of insects [19–21], while passive dispenser uses solid-matrix rubbers [22], polyethylene (PE) or polyvinyl chloride (PVC) tubes [23], or wax drops [24]. They have been used in the management of some lepidopteran insects, such as *Cydia pomonella* (L.) [25], *Grapholitha molesta* (Busck) [26], and *Lobesia botrana* (Denis and Schiffermuller) [20, 27]. So far, the MD reported for *T. absoluta* has all used passive dispensers [28–31].

The major mechanisms for MD are competitive and non-competitive [32, 33]. This study conducted tests on a passive dispenser with various dosages and its distribution densities in the greenhouse tomatoes, in order to understand the mechanism of MD on *T. absoluta* and to determine the efficacy of MD. The efficacy of the active aerosol dispenser on the MD was also tested.

## Results

### *Tuta absoluta* attractiveness of PE dispensers containing different dosages of sex pheromones

**Fig. 1:**
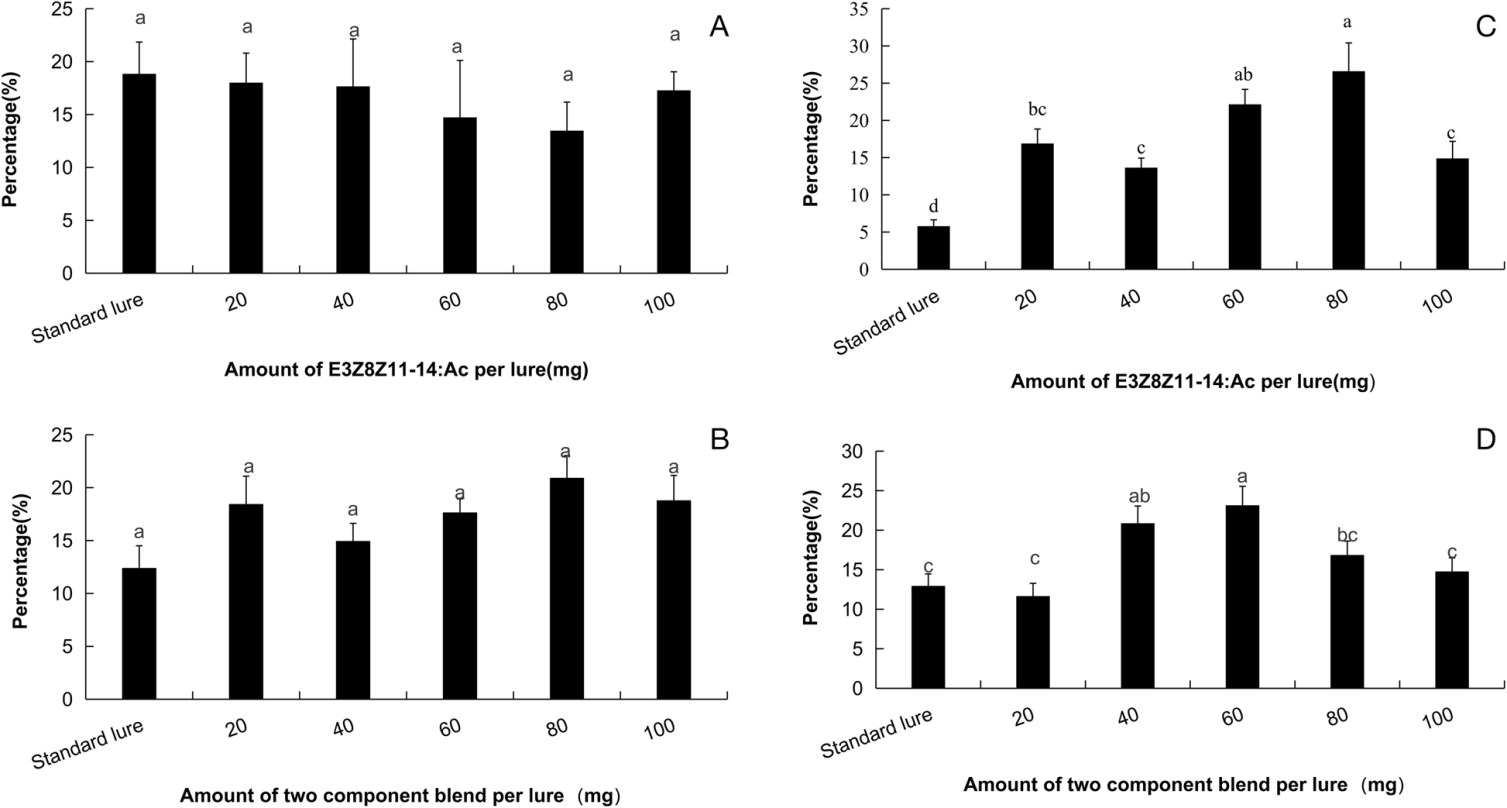
Percentage of moth caught in traps with standard lure and sex pheromone dosages containing single or two components in the dispensers for MD in greenhouse tomatoes **A** containing single component in Xichang; **B** containing two components in Xichang; **C** containing single component in Chifeng; **D** containing two components in Chifeng. Bars in the same subfigure with different letters are in significant difference at *P*<0.05

To determine the appropriate dosage of the single component *E*3*Z*8*Z*11-14:Ac and binary combinations of *E*3*Z*8*Z*11-14:Ac and *E*3*Z*8-14:Ac in dispensers for MD, we tested the attractiveness of dispensers to *T. absoluta* males in Xichang, where the adult population density was high, and in Chifeng, where the density was low. In Xichang, there was no difference in the attractiveness of PE dispersers containing different dosages of a single component in the sex pheromone, compared to that of standard monitoring lure (*F=*0.34*, df=*29*, P*=0.881) (Fig. 1A). Differences among the attractiveness in the binary mixtures with different dosages in the MD dispensers and the standard monitoring lure were also not significant (*F*=2.04, *df*=28, *P*=0.11) (Fig. 1B). In Chifeng, the attractiveness was all greater in the single component dispenser with all the 5 dosages, in comparison with that in the standard monitoring lure (*F*=10.39, *df*=29, *P*<0.01) (Fig. 1C). The number of moths attracted in the trap containing 80 mg (AI) was 20.8% greater than that in the monitoring trap, while the numbers in the 60 and 80 mg were similar (Fig 1C). For the binary mixture, the 80 and 60 mg dispensers attracted 8.1% and 5.8% more moths, respectively, compared to the monitoring lure (*F*=5.44, *df*=29, *P*<0.01), whereas dispensers containing other dosages and the monitoring lure had the similar attractiveness (Fig. 1D)

### Sex pheromone dosage in dispensers on the MD of *T. absoluta*

**Fig. 2:**
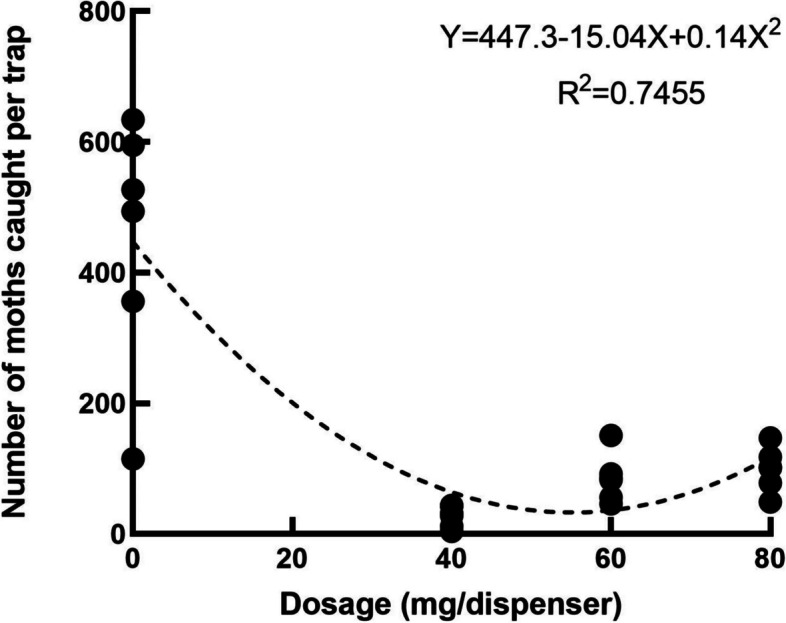
Relationship between numbers of *T. absoluta* moth catches in monitoring traps and sex pheromone dosages in the dispensers for MD in greenhouse tomatoes

Results showed that the numbers of moths caught in monitoring traps in the treatment greenhouses with the PE dispensers containing *E*3*Z*8*Z*11-14:Ac at all the dosages was respectively lower than that in the control greenhouse (*F*=21.63, *df*=29, *P*<0.01) (Fig. 2). There was a significant negative relationship between the pheromone dosage in the dispensers and the number of moths caught in the monitoring traps (*R*=-0.7944, *P*<0.01) (Fig. 2).

The pheromone dosages in the PE dispensers significantly decreased the degree of damage to the leaves caused by *T. absoluta* (*F*=3.67, *df*=19, *P*=0.03), and the damage in the control greenhouse was significantly higher than that in the treatment with MD (Table 1). Pheromone dosages in the dispensers also significantly reduced the number of larval *T. absoluta* (*F*=6.198, *df*=19, *P*=0.004) (Table 1). The results showed that the number of damaged leaves and larval populations increased dramatically in the control greenhouses, but it remained low in the treatment greenhouses. The leaf damage rate of the control greenhouse without dispensers increased by 251.1±62.4%, and the number of larvae increased by 332.1±95.5%. The damage rate in the greenhouse treated with dispensers containing *E*3*Z*8*Z*11-14:Ac at 40, 60, and 80 mg increased by 23.1±24.0%, 128.1±80.9% and 65.5±39.4%, respectively, whereas the damage rate in the greenhouse treated with dispensers containing 80 mg of binary mixture decreased by 2.3±19.7%. The corresponding number of larvae increased by 23.4±5.6% and 47.0±50.1%, decreased by 5.9±12.0%, and increased by 25.0±62.9%, respectively.

**Table 1:** Effects of mating disruption with different dosages of sex pheromones on tomato leaf damage and larval *T. absoluta* populations

AI per dispenser	mg per dispenser	Increase in the leaves damaged (%)	Increase in larval population (%)
0	0	**+** 251.1±62.4a	**+** 332.1±95.5a
*E*3*Z*8*Z*11-14:Ac	40	**+** 23.1±24.0b	**+** 23.4±5.6b
*E*3*Z*8*Z*11-14:Ac	60	**+** 128.1±80.9b	**+** 47.0±50.1b
*E*3*Z*8*Z*11-14:Ac	80	**+** 65.5±39.4b	- 5.9±12.0b
Binary blend of *E*3*Z*8*Z*11-14:Ac and *E*3*Z*8-14:Ac	80	- 2.3±19.7c	**+** 25.0±62.9b

The numbers in the same column followed by different letters are significantly different at *P* < 0.05.

### PE dispenser density and aerosol dispenser effects on the mating disruption of *T. absoluta*

**Fig. 3:**
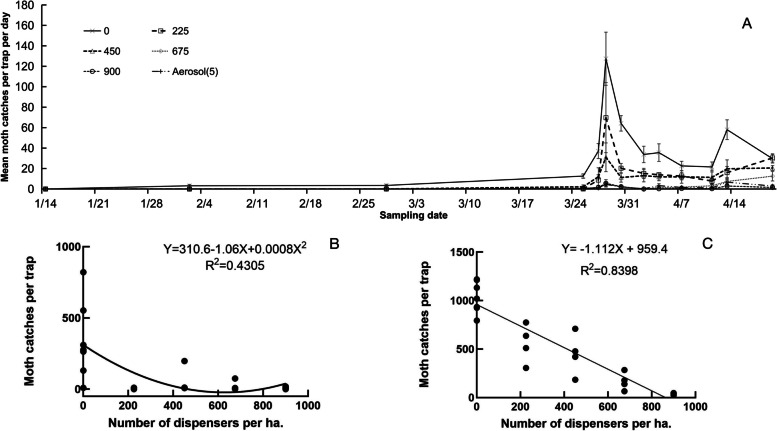
Suppression effect of sex pheromone dispenser density on *T. absoluta* population dynamics and relationship between the density and adult numbers **A** on population dynamics; **B** relationship with low adult numbers (<10/trap/day); **C** relationship with high adult numbers (>10/trap/day)

When the population density of adult *T. absoluta* in the greenhouse was low (< 10/trap/day), the number of moths caught decreased with increasing of density of dispensers (Fig. 3A). There was a significant negative curvilinear relationship between the density of sex pheromone dispensers and the number of moths caught in the greenhouses (*R*=-0.71, *P*<0.01) (Fig. 3B). When the population density of the moths in the greenhouses was high (>10/trap/day) in the greenhouses, the number of moths trapped decreased strikingly with increasing dispenser density (Fig. 3A), and the linear relationship between the dispenser density and the number of moths was more negative (*R*=-0.97, *P*<0.01) (Fig. 3C).

**Fig. 4:**
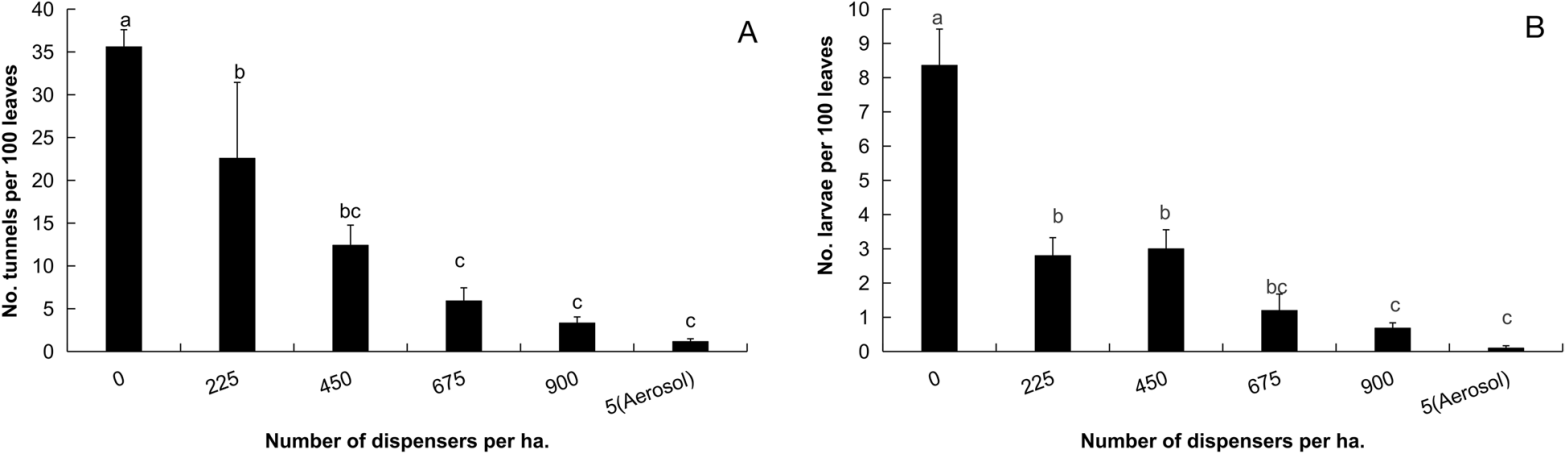
Suppression effect of sex pheromone dispenser density on number of larval tunnels and numbers of *T. absoluta* larvae in tomato leaves. **A** on number of larval tunnels; **B** on numbers of larvae. Bars in the same subfigure with different letters are in significant difference at *P*<0.05

Mating disruption at densities of 225, 450, 675, and 900 dispensers per ha decreased the number of larval channels in tomato leaves by 36.5%, 65.2%, 83.4%, and 90.7%, respectively, in the treatment greenhouse compared with the control greenhouse (*F*=13.27, *df*=123, *P*<0.01; Fig. 4A). MD by the aerosol dispensers at a density of 5 per ha reduced the number of larval channels by 95.2%, while the reduction in the number of larval channels caused by the aerosol dispenser was similar to those treated by the PE dispensers at the density of 450, 675 or 900 (Fig. 4A).

Compared with that in the control greenhouse, the number of laval *T. absoluta* in the tomato leaves in the treatment greenhouse was suppressed by 66.3%, 63.9%, 85.5%, and 91.6%, respectively, by the PE dispensers at densities of 225, 450, 675, and 900 per ha (*F*=14.27, *df*=123, *P*<0.01; Fig. 4B). The number of larvae in tomato leaves decreased by 98.7% in the greenhouses treated with the aerosol dispensers at a density of 5 per ha (Fig. 4B). However, the suppression effects on larval number were similar among the PE dispersers at densities of 675, 900, and the aerosol dispenser (Fig. 4B)

## Discussion

This study compared the field trapping of passive PE tube dispensers at different dosages and the adult *T. absoluta* attraction in tomato greenhouses. When the adult population density was low, the moth-attracting amount of single E3Z8Z11-14:Ac and binary mixtures at a dose of 60-80 mg was the highest (Fig. 1B and D), but when the adult population was high, there was no significant difference among different dosages (Fig. 1A and C). Moreover, in the MD tests, the results showed that the effect of different dosages of PE dispensers on the MD was not significant (Table 1). The setting density of the 80 mg PE dispensers significantly affected the amount of moth catches in the greenhouse. Under low-density adult conditions, the correlation curve showed typical competitive mating disruption, whereas under high- density adult conditions, the relationship was a linear (Fig. 3B and C).

Competitive MD is closely related to the density of sex pheromone dispensers [33, 34]. This study showed that, when the density of the *T. absoluta* adult population was low, a setting of 225 PE dispensers per ha had some effect on the suppression of the population, however, when the population density was high, such a setting could not achieve a high disruption rate (Fig. 3A), and the control efficacy on the damage rate and number of *T. absoluta* larvae was also limited (Fig. 4). A density of 675-900 dispensers per ha was required to achieve a high control result (Fig. 4).In contrast, the impact of sex pheromone dosage in the PE dispenser had not showed dramatic difference (Table 1), which is similar as reported in the literature [30, 31, 35]. The increase in the density of dispensers not only increased the cost of material, but also added the additional labor cost for tomato growers.

This study also confirmed that the active aerosol dispenser of non-competitive MD was not affected by the density of adult *T. absoluta*. The active aerosol dispenser released 6 μl of mixture each time, containing about 300 μg active compounds of the sex pheromone. Such a high dosage inhibited the release of female sex pheromones and disrupted the mating of *T. absoluta* adults, even if the density of adults was relatively high (Yongjun Du, unpublished data). Therefore, even if the disruption rate is not the greatest (Fig. 3A), the damage rate and the number of larvae were the lowest (Fig. 4). Only one aerosol dispenser was needed in a greenhouse of less than 0.13 ha, because the calling period of *T. absoluta* females is only 4-5 hours in the early morning [16], so the release of sex pheromones in the dispenser was set to 7 hours per day. In this way, one set of aerosol dispensers can be released more than 25,000 times, covering two tomato growing seasons. In the competitive mating disruption, 675-900 PE dispensers (each with a dosage of 80 mg) per ha were needed, and the effective period was only for one tomato growing season. In terms of per unit cost, the PE dispenser is higher. However, it has been reported that active aerosol dispenser was not effective on mating disruption against some insect species. For example, it was effective against *G. molesta*, but not effective against *C. pomonella* in the same orchard in Michigan, USA [26]. The reason might be related to the solubility and volatility of sex pheromone compounds. The solubility and volatility of sex pheromone compounds *E*8*E*10-12:OH of *C. pomonella* are relatively poor, especially when the temperature is low in the field. The solvent solubility and volatility of *E*7*Z*9-12Ac of *L. botrana* [20, 27], Z11-16:Ald of *Chilo suppressalis* (Walker) [36] and *Z*11-16:Ac of *Sesamia inferens* (Walker) [37] are relatively good.

## Conclusion

This study demonstrated that the passive PE dispensers are typical competitive mating disruption and that the density of adult *T. absoluta* is an important factor. MD dispensers should be set up at low density or when the young tomato seedlings are transplanted. A total of 900 passive PE tubing dispensers loaded with 80 mg sex pheromone per ha or one active aerosol dispensers per greenhouse were required to effectively control *T. absoluta*.

## Materials and methods

### Experimental materials and sites

The sex pheromone lures, winged sticky traps, polyethylene (PE) tubing and active aerosol dispensers used in the experiment were all made by NewCon Inc. (Ningbo, China). The aerosol dispenser (5% a.i. *E*3*Z*8*Z*11-14:Ac and *E*3*Z*8-14:Ac (9:1)) started to spray at 02:00 AM, and ended at 09:00 AM, and the interval between sprays was 6 min. Each time it released 6 μl.

The experiment was conducted in greenhouses from January 13 to April 20, 2024 in Ningcheng, Chifeng, Inner Mongolia Autonomous Region (41.59 N, 122.99 E) and in Youjun Town, Xichang City, Sichuan Province (39.91 N, 116.39 E). The area of each of the test greenhouses was 0.1 ha. In Xichang, the distance between greenhouses within the same row was 1 m, and the distance between different rows was 8-10 m. The greenhouses were ventilated during the day. In Chifeng, the distance between greenhouses in the same row was 5-6 m, and the distance between different rows was 12-15 m. The greenhouses were also ventilated during the day. Two neighboring test greenhouses were separated by a greenhouse. The test greenhouses at both sites were routinely managed according to the local grower standard.

### *Tuta absoluta* attractiveness of PE dispensers containing different dosages of sex pheromones

At both experimental sites, PE tube dispensers containing 20 mg, 40 mg, 60 mg, 80 mg, and 100 mg of single component *E*3*Z*8*Z*11-14:Ac or dual-components *E*3*Z*8*Z*11-14:Ac and *E*3*Z*8-14:Ac (9:1) of *T. absoluta* sex pheromones were respectively prepared, and the standard lure for monitoring (*E*3*Z*8*Z*11-14:Ac and *E*3*Z*8-14:Ac 9:1 ratio, the dosage is 1 mg per PVC tubing lure) was used as the control. Winged sticky traps, each with one dosage of the PE tube dispenser, were placed along the middle-row of tomatoes in a greenhouse. The distance between the traps was 10 m. Height of the trap was 30 cm over the ground. Number of *T. absoluta* adults in each of the trap was recorded daily. The sticky bottom was replaced when the adult number in the trap was large. The trapping experiment was replicated five times at each experimental site.

### Sex pheromone dosage in dispensers on the MD of *T. absoluta*

On March 27 - April 22, in the fruiting stage of greenhouse tomatoes in Youjun town, Xichang, PE tube-dispensers containing 40 mg, 60 mg, and 80 mg of single component *E*3*Z*8*Z*11-14:Ac, and 80 mg binary mixture of *E*3*Z*8*Z*11-14:Ac and *E*3*Z*8-14:Ac (9:1) were tested for MD in greenhouses. 40 dispensers of each single- or binary-component dosage were evenly distributed in one greenhouse. The height of the dispenser was about 50 cm over the ground. One monitoring trap with the PVC tubing lure was placed in the center of each treatment greenhouse.

To determine the degree of damage to tomato plants caused by *T. absoluta*, five sampling sites were respectively selected from the east, west, south, north and center of each treatment or control greenhouse. Ten plants were checked at each site, and the number of damaged leaves was recorded. Two leaves were randomly selected from the top, middle and bottom of each tomato plant. The number of damaged leaves and the number of larvae on each leaf were recorded.

The number of trapped moths was counted every 2 days, while the number of damaged leaves and the number of larval *T. absoluta* in damaged leaves were checked every 5 or 7 days. There was no dispensers but a monitoring trap in the control greenhouse. The dosage test was replicated 4 times, each in a greenhouse.

### PE dispenser density and aerosol dispenser on mating disruption of *T. absoluta*

The experiment was conducted in greenhouses in Youjun Town from January 13 to April 20, 2024, when the tomatoes were in the flowing stage. A total of 4 densities of PE tube dispensers (225, 450, 675, 900 dispensers/ha), all with the dosage of 80 mg per dispenser, and the aerosol dispenser for MD (1 in each greenhouse) were tested for *T. absoluta* MD. The dispensers at each density were evenly distributed in a greenhouse for the test. A *T. absoluta* monitoring trap with the sex pheromone lure was placed in the center of each treatment or control greenhouse. There was no dispenser but a monitoring trap in the control greenhouse. The experiment was replicated five times, each in a greenhouse. The method and frequency to determine foliar damage and larval number were the same as those described above.

### Statistical analysis

The data were analyzed via SPSS 17.0. The resulting data of *T. absoluta* adults, larvae and the damage were analyzed via one-way ANOVA. Pairs of treatment means were compared and separated by the Duncan's multiple range test. The Pearson correlation method was used to determine the relationship between the number of moth catches and the sex pheromone dosage or the dispenser density.

## Data Availability

The data that support the findings of this study are available upon reasonable request.
